# Mr. Simpson's Case of Oscheocele

**Published:** 1805-06-01

**Authors:** Andrew Simpson

**Affiliations:** Surgeon. Newtown Stewart, Galway


### *To the Editors of the Medical and Physical Journal.*

Gentlemen,

As progressive improvement can only be generally beneficial by public declaration and faithful reduction to practice, if the following observations and practical statement deserve public attention, you will decide accordingly.

By

By the individual and collective endeavours of chirurgeons, improvements have fortunately and progressively augmented the melioration of manual curative intention, and happily diminished the previously unavailing and indeterminate application of instruments. These generally applicable observations being premised, I transmit you my attentions to, and successful treatment of an instance of oscheocele.

It occurred in a lad, seventeen or eighteen, of radically imbecile systematic composition and conformation, but generally enjoying tolerable health, excepting occasional transitory inconvenience from hernia congenita scrotalis, which had frequently appeared from earliest infancy, but was hitherto readily reducible by his efforts, especially when horizontally reclined, &c. The strangulation of the prolapsed contents occurred about the 18th or 19th of June, 1799. Upon the 20th I was desired to visit him, after several fruitless endeavours were employed for its reduction, &c. The afflicting and designating symptoms were principally the following: general distressing uneasiness, pulse varying betwixt 100 and 120, tolerably regular, somewhat sharply impressing the finger; breathing anxious and frequent; visage narrow, contracted and rather squalid; eyes expressive of sorrowful conceptions, depressed in the cavities; he was distressed with continual pervigilium, restlessness, and frequent propensity to change his position; violent abdominal pains with successive frequent hiccup; likewise anorexia, nausea and vomiting; which last generally produced bilious matter, mucous gastric excretion, and the liquids swallowed, and an insatiate desire for drinking, &c. The *prolapsus hernialis* resembled a globular figure, (capable of holding 12 oz. of liquid) tensely elastic, painful, slightly inflamed, and possessed an obscure degree of transparency; suppression of intestinal depletion likewise occurred, but urinary secretion was sufficiently evacuated. An endeavour by manual application was ineffectually attempted for the reduction of the hernial contents. In gradual succession, all the ordinary medicinal and remedial expedients customarily employed in similar disorders were assiduously administered, astringent frigerating epithemata of diversified composition and application, especially solutions of acetis plumbi, ammonia muriata, spiritus vinosus gallicus aqua et acetum, in equal portions, &c.; the tumour was regularly surrounded with pieces of linen moistened in the specified liquid, but apparently without discernible advantage. The buttocks were preserved in an elevated position to favour the ingress of the the hernial protrusion, venesection to produce deliquium, the patient being placed in an erected posture during the operation, but without the intended consequence, although a considerable depletion was procured at each repetition. Frequently repeated attempts were fruitlessly employed at intervals, to return the protruded contents. Purgative compositions were exhibited internally without apparent advantage; stimulating injections; likewise one composed of infusion of nicotiana (ʒi. to ℥xij. of water) not possessing the apparatus for preparing and injecting the clysma fumosum, was exhibited, but seemingly produced no perceptible advantage, apparently increasing the nausea and the abdominal uneasiness. Immersion in the balneum tepidum was employed, and thereafter unsuccessful endeavours for the reduction. Anodyne preparations were administered to procure composure, relieve pain, and produce general relaxation; which appeared to conduce to temporary alleviation of pain.

Grievously disappointed in all former endeavours, I again repeated the old fashioned, but frequently beneficial method of inverting the patient over the shoulders of an assistant; and when suspended in that situation, the head and shoulders being supported by his father, I made another unavailing experiment for reduction; fortunately at that moment, when expectations and remedial employ, save the operation, were considered inadequate, an imaginary conception suggested the probable advantage that might be obtained from giving the body a sudden elevation and depression; I therefore directed the assistant, (a stout firm man) supporting the extremities, to spring forcibly from the floor, and descend with a bounding exertion, (hic mihi equidem vix sunt verba idonea) while the assistant, supporting the shoulders, &c. retained the patient as much as possible in a proper direction for relaxing the abdominal muscles; during which evolutions I retained manual pressure upon the tumour; when, after repeated gradually and cautiously increased movements of the assistant, we obtained the pleasing satisfaction of observing the hernial extrusion speedily subside, and ultimately disappear. Immediately he experienced material and welcome relief from the most distressing symptoms. The oscheocele had been of five or six days duration; a violent diarrhœa succeeded, and in the course of twenty-four hours had about thirty intestinal discharges, originating from the carthartic medicaments and enemata which were employed. He quickly recovered, and by the applications of a proper elastic bandage, no further descent scent occurred during his residence here. He went to the West Indies in the ensuing autumn, and, by information, I understood he continued free from his complaint.

*Newtown Stewart, Galway,* ANDREW SIMPSON, Surgeon.

*April 2,* 1805.

